# The prodromal individuals’ perspective on active recruitment for early detection of α-synucleinopathies

**DOI:** 10.1038/s41531-025-00979-0

**Published:** 2025-05-09

**Authors:** Sinah Röttgen, Eva Schaeffer, Annette Rogge, Ramona Hartung, Konstantin Kufer, Gereon R. Fink, Daniela Berg, Anja Ophey, Michael Sommerauer

**Affiliations:** 1 Cognitive Neuroscience, Institute for Neuroscience and Medicine (INM-3), Research Centre Juelich, Juelich, Germany; 2 Department of Neurology, Faculty of Medicine and University Hospital Cologne, University of Cologne, Cologne, Germany; 3 Department of Neurology, University Hospital Kiel, Christian-Albrechts-University Kiel, Kiel, Germany; 4 Department of Neurology, Nordseeklinik Helgoland, Helgoland, Germany; 5 German Centre for Neurodegenerative Diseases (DZNE), Bonn, Germany; 6 Centre of Neurology, Department of Parkinson, Sleep and Movement Disorders, University of Bonn, Bonn, Germany; 7 Department of Medical Psychology | Neuropsychology and Gender Studies, Centre for Neuropsychological Diagnostics and Intervention (CeNDI), Faculty of Medicine and University Hospital Cologne, University of Cologne, Cologne, Germany

**Keywords:** Movement disorders, Neurodegenerative diseases, Parkinson's disease

## Abstract

Only the timely detection of individuals with incipient α-synucleinopathies can pave the way for developing disease-modifying therapies. Our aim was to explore the views of individuals with isolated REM sleep behavior disorder (iRBD), actively recruited from the general population, on the ethical justifiability of active recruitment and their experiences with risk disclosure. This mixed-methods study surveyed individuals with iRBD, confirmed by video-polysomnography, utilizing an interdisciplinary-developed online questionnaire. Of 99 invited individuals, 75 (75.8%) answered the survey. While 55.6% experienced the information on the increased risk as burdensome, 63.9% supported risk disclosure if consent had been obtained beforehand. Almost all individuals (96.2%) regarded our active recruitment method as appropriate, and 86.7% indicated they would participate again. Open-text responses indicated that key motivations included access to information and care, and contributing to research progress. This well-received recruitment strategy could serve as a model for future screening initiatives in α-synucleinopathy research.

## Introduction

Parkinson’s disease (PD), dementia with Lewy bodies (DLB), and multiple system atrophy (MSA) are recognized as α-synucleinopathies due to their shared pathology involving the accumulation of misfolded α-synuclein^1^. This process starts years or decades before the clinical diagnosis, progressing through a “prodromal phase” with only subtle clinical signs during incipient neurodegeneration^2^. This early time window presents a time window for effective, targeted interventions slowing down or halting neurodegenerative processes^3,4^. The reliable and early identification of affected individuals is not only key to conduct neuroprotective trials at the right time but it is an essential prerequisite to develop such treatments, as they can only be evaluated within the corresponding target population. Recent advances in detecting pathological α-synuclein mark a significant milestone as they open new avenues for early identification of affected individuals^5^. In the near future, the screening for seeding-competent α-synuclein species may even allow for a biological diagnosis at a particularly early stage^6^. However, early identification creates an ethical dilemma: on the one hand, recruitment of large cohorts is essential for the development and testing of targeted, neuroprotective therapies, yet on the other hand, no such therapies can currently be offered. Due to the current lack of pharmacological disease-modifying treatments and the uncertainty if and when burdensome symptoms may break through in a clinically “prodromal individual”, early detection presents substantial ethical challenges^7^.

Generally, experts recommend individualized risk disclosure^8,9^. Still, current recommendations are cautious regarding open risk communication in early α-synucleinopathies^10,11^. This aligns with a survey of individuals with diagnosed PD on their view on early risk disclosure before overt PD symptoms: most were skeptical on being informed in a prodromal phase if the information was not accompanied by the offer of a disease-modifying therapy^12^.

Isolated REM sleep behavior disorder (iRBD) is a highly specific indicator for an incipient α-synucleinopathy^13^, with >90% risk of phenoconversion to PD, DLB, or MSA within 15 years following iRBD diagnosis^14^. Individuals with iRBD can reliably be identified through video-polysomnography due to the missing muscle atonia and characteristic movement patterns observed during REM sleep. In rarer cases, affected individuals independently seek medical attention, either due to self-inflicted injuries during sleep or disturbing their bed partner. However, in most cases, the condition remains unreported by the affected individuals and undetected in routine medical practice. Active recruitment in this context would create a unique opportunity to establish larger prodromal cohorts and may constitute a blueprint for recruiting biologically defined cohorts.

Although discussions about risk disclosure in iRBD are becoming more prominent, they are primarily conducted within the scientific community, typically without meaningful input from those affected. In clinical practice, most clinicians perform risk disclosure in individuals with iRBD seeking help for their sleep disorder^8,9,15^. Regarding the decision to disclose the risks associated with an iRBD diagnosis, Stefani and colleagues argue that the limited direct clinical benefits should be taken into account^11^. As risk disclosure could lead to adverse psychological effects, Sixel-Döring and colleagues advised against an unconditional risk disclosure to all individuals with iRBD^10^. Teigen and colleagues summarized interviews with 44 physicians with expertise in sleep neurology, and 93.2% informed most of their patients with iRBD on the risk of phenoconversion, but only a third consistently obtained prior consent to reveal this information^9^. However, there is a critical gap in understanding how those at risk perceive and process the risk disclosure, especially if they were not aware of their condition and its implications.

Risk disclosure strategies may vary significantly depending on whether individuals with iRBD are already seeking medical support due to their sleep disorder (typically following own prior information-seeking about the condition). Studies in individuals with iRBD seeking healthcare support for their sleep disorder reported a strong preference for also receiving prognostic information^16,17^. A proactive identification strategy as part of a research project contrasts to this as individuals are unaware of their RBD and any relevant neurodegenerative condition, necessitating a more intentional approach of discussing risks with participants before enrollment. Surprisingly, despite active recruitment of iRBD cohorts (and additional prodromal cohorts), there is a lack of data on the individuals’ perspective regarding whether and how appropriate risk disclosure should be conducted.

The primary objective of this study was to understand the participants’ needs and experiences with risk disclosure in an active cohort recruitment setting for detecting early α-synucleinopathies. Precisely, this study aimed to answer whether *active* recruitment of individuals with iRBD from the general population is ethically justifiable. Secondary goals were to identify the individuals’ motivation for participation, learn about their experiences, and establish recommendations for active cohort recruitment in early α-synucleinopathy research.

## Materials and methods

### Participants

From July 2020 to November 2023, individuals with iRBD were actively identified through a structured approach, beginning with newspaper advertisements for a research project on sleep disturbances^18^. During a first telephone contact with the study team and before polysomnography, all interested individuals were informed about the possibility of being eventually diagnosed with iRBD, including education about the risk of ~80% of individuals with iRBD advancing to a neurodegenerative disorder such as PD within 15 years. This information allowed for early withdrawal before any data was obtained, if individuals preferred not to receive such information. The screening process included a structured telephone interview followed by an evaluation with video-polysomnography evaluated by a sleep specialist^18^. Upon confirmation of iRBD, participants were informed of their diagnosis and underwent annual clinical follow-ups.

Inclusion criteria for participants with iRBD were age 50 to 80 years, polysomnography-confirmed iRBD following the International Classification of Sleep Disorders III criteria^19^, and the ability to give informed consent. For further details, please refer to Seger et al. ^18^. Individuals identified with iRBD who have already had at least one clinical visit were invited to participate in the survey in November 2023.

### Standard protocol approvals, registrations, and patient consents

The study received approval from the local ethics committee of the Medical Faculty at the University of Cologne. Written informed consent was obtained from all participants.

### Study and survey design

In this exploratory mixed-methods study, we designed a semi-structured online questionnaire to understand the preferences of individuals with iRBD on risk disclosure (Supplementary Fig. S1). We designed the questionnaire using a step-wise approach, incorporating several correction loops after feedback from both scientific experts and two individuals from the iRBD cohort.

The questionnaire comprised 20 questions. Fourteen were single-choice questions; for two we used a multiple-choice format. All these questions included the option “I cannot/do not want to answer this question”. An additional four optional open-answer questions allowed the participants to provide more detailed information on their answers. Moreover, we assessed symptoms of depression and anxiety as well as resilience using the depression scale of the Patient Health Questionnaire (PHQ-9)^20^, the Generalized Anxiety Disorder 7 (GAD-7)^21^, and a modified four-point version without a neutral midpoint of the Brief Resilience Scale (BRS)^22^, respectively.

The survey was implemented and distributed using SoSci Survey^23^ (www.soscisurvey.de). Participants were once reminded four weeks following the first invitation.

### Data analysis

Statistical analyses were conducted using IBM SPSS Statistics version 28.0 (IBM Corp, Armonk, New York, United States of America) and R Studio. For single-choice and multiple-choice questions, the distribution of response categories was reported (*n*, %). Data normality was assessed with Shapiro-Wilk and Kolmogorov-Smirnov tests and Q-Q plots. For the PHQ-9 and GAD-7, total scores were calculated as the sum of item responses. Resilience scores were computed as the mean of item responses. As the data were not normally distributed, Spearman’s rank correlations were applied to assess the association between clinical scores and subjective burden after risk disclosure.

Additionally, we used conventional content analysis to obtain a more in-depth understanding of the participants’ experiences and attitudes toward risk disclosure^24^. Answers to the open-answer questions were analyzed and grouped into overarching categories derived from data codes by two psychologists (SR and AO) with expertise in iRBD research.

## Results

During our screening efforts, *n* = 886 individuals responded to the newspaper advertisements, of whom *n* = 229 individuals were invited for video-polysomnography. The remaining *n* = 657 individuals dropped out before video-polysomnography. Among these individuals, only two (0.30%) explicitly withdrew from the screening process after being informed about the possibility of eventually being diagnosed with iRBD and the disclosure of the accompanying risk. Main reasons for not proceeding with the study protocol were the presence of exclusion criteria and non-response to the inquiries.

From the polysomnography-confirmed individuals with iRBD, we invited *n* = 99 individuals with at least one clinical visit to participate in the mixed-methods survey, with *n* = 75 (75.8%) responding. Seventy-two (96%) datasets were included for the final analysis: Three were excluded due to missing data or phenoconversion to PD. Up to 98.6% responded to optional open-answer questions. Demographic and clinical characteristics are presented in Table 1. Most participants reported no relevant symptoms of anxiety or depression and had normal to high resilience.

**Table 1:** Demographic and clinical characteristics

	Mean	Range	Threshold
Age [years]^a^	68.97 ±6.35	55–82	
Sex (male:female)^a^	58:11		
Symptom duration [years]^b^	9.63 ±6.74	1–32	
Time since diagnosis [years]^c^	1.75 ±1.18	0–3.25	
BRS mean	3.72 ±0.85	1.5–5	
Low resilience			14.08%
Normal resilience			60.56%
High resilience			25.35%
PHQ-9 total^d^	3.70 ±4.26	0–18	
None-minimal depression			72.86%
Mild depression			17.14%
Moderate depression			7.14%
Moderately severe depression			2.86%
GAD-7 total^d^	2.27 ±3.12	0–17	
Minimal anxiety			88.57%
Mild anxiety			8.57%
Severe anxiety			2.86%

If not specified, *N* = 72. Results are expressed as mean, ± SD, (range), % within a questionnaire cut-off or absolute numbers. Symptom duration is the self-reported duration of iRBD symptoms. *BRS* Brief Resilience scale, *PHQ-9* Patient Health Questionnaire, *GAD-7* Generalized Anxiety Disorder.

^a^*n* = 69.

^b^*n* = 67.

^c^*n* = 68.

^d^*n* = 71.

### Relevance of sleep disorder and opinions on risk disclosure

**Fig. 1:**
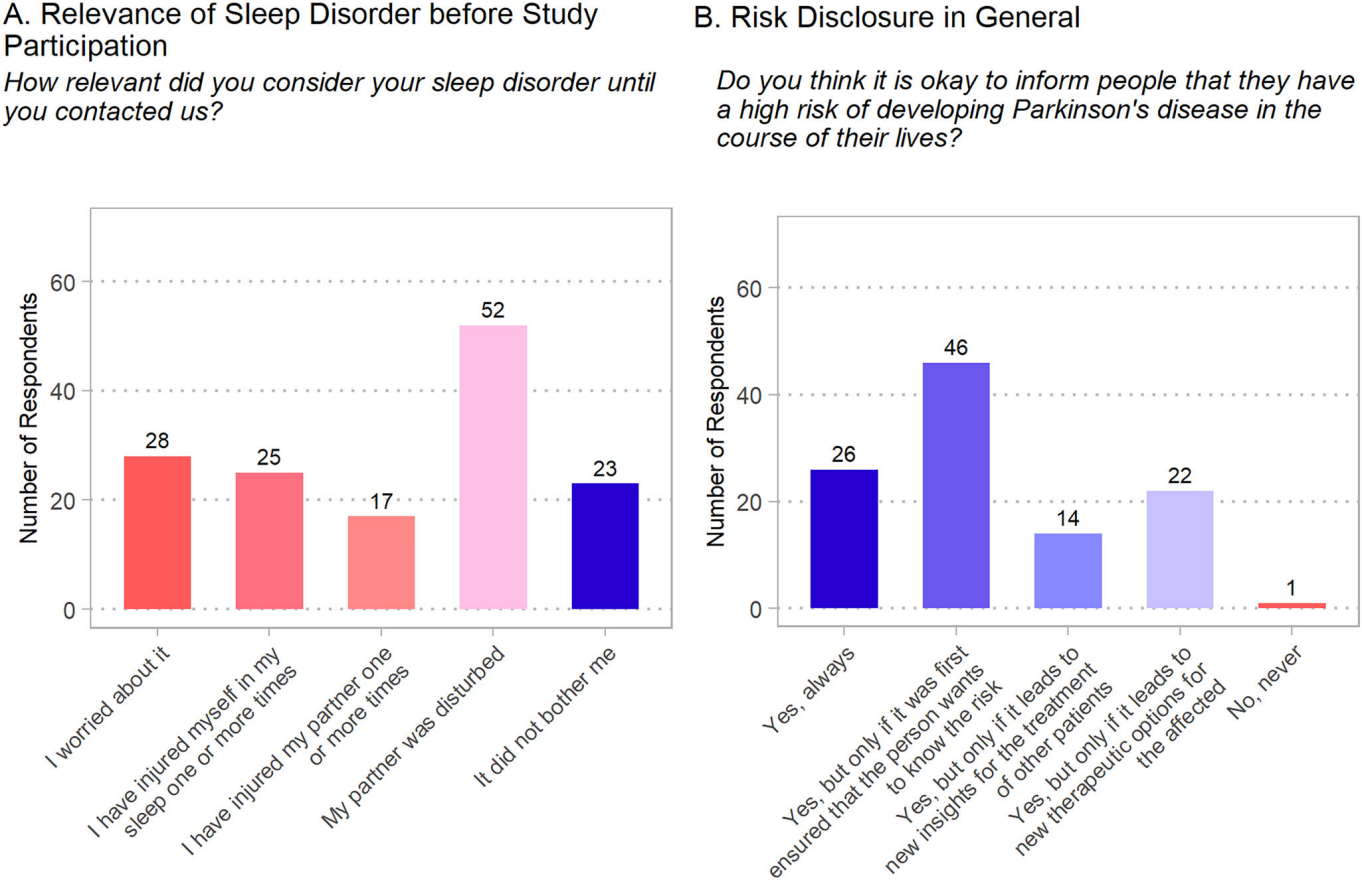
Opinion of individuals with iRBD on the relevance of their sleep disorder before study participation and on risk disclosure in general. **A** How participants rated the relevance of their REM sleep behavior disorder before study participation. **B** Participants’ general attitudes towards risk disclosure. Both items were multiple choice items. Displayed are the absolute numbers of responses for each answer option.

Participants were asked, *“How relevant did you consider your sleep disorder until you contacted us?”* to explore the clinical relevance and impact of the disturbed sleeping habits. Only about a third (38.8%) worried about their sleep behavior, despite 72.2% disturbing their bed partners, 34.7% having injured themselves, and 23.6% having injured their bed partners (Fig. 1A).

Only one participant (1.39%) did not to support risk disclosure when asked “*Do you think it is okay to inform people that they have a high risk of developing Parkinson’s disease in the course of their lives, even before they show clear signs of the disease?*”. Two-thirds favored risk disclosure if it was ensured beforehand that the person wanted to know about the risk (Fig. 1B).

**Fig. 2:**
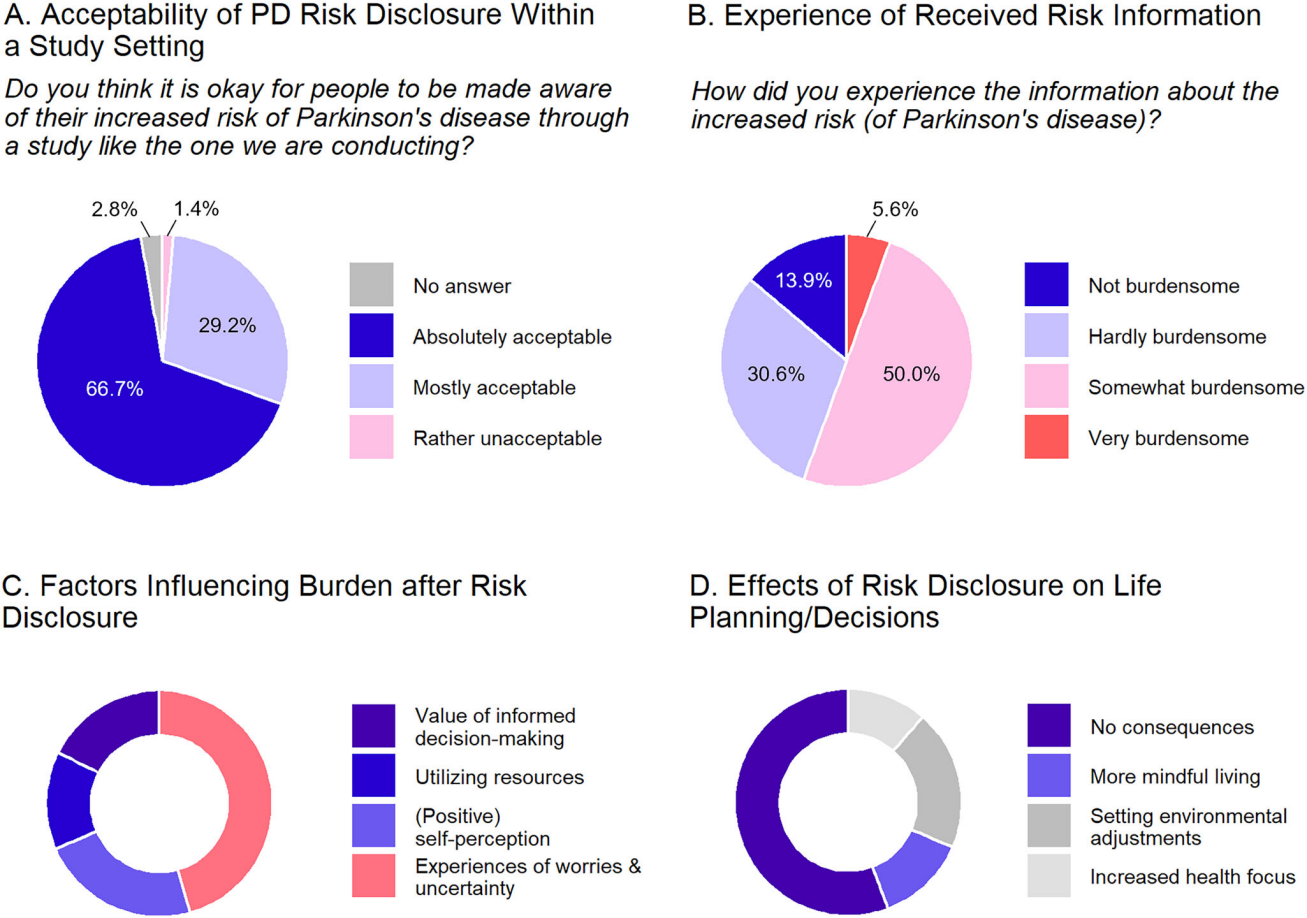
Opinion of individuals with iRBD on and experiences with risk disclosure in an active recruitment setting. **A** Participants’ views on the acceptability of risk disclosure in the active recruitment setting used during their study enrolment. **B** How participants experienced receiving risk disclosure. **C** Factors that influenced whether participants experienced burden or no burden following risk disclosure. **D** Effects (or lack thereof) that risk disclosure had on participants’ life planning and decision-making. Proportions in the donut chart correspond to the number of responses in each category. More information and exemplary quotes for each category can be found in Tables S1 and S3.

Despite one-third not perceiving their disorder pathological, 95.9% viewed our recruitment approach and the associated risk disclosure as appropriate or mostly okay, while one person (1.4%) disagreed (Fig. 2A).

### Experiences with risk disclosure

Although screening was highly accepted, 55.6% experienced risk disclosure as very or somewhat burdensome, while the others reported finding it hardly or not burdensome (Fig. 2B). We examined whether specific individuals might be at particular risk for not coping well with risk disclosure due to a combination of depressive symptoms, anxiety, and low resilience. Interestingly, subjective burden was not correlated with psychological well-being (all │*r*│ < 0.20, all *p* > 0.10) and most participants reported good psychological well-being (Table 1, Supplementary Fig. S2).

A total of *N* = 229 free-text responses provided insights into participants’ concerns motivations. When participants experienced the risk disclosure as burdensome, this was often associated with worries and feeling uncertain about future outcomes (41.9%; a total of *n* = 61 answers were given to this question, Fig. 2C, Supplementary Table S1). For instance, one participant shared that the disclosed risk was burdensome since the *“disease is not curable”*. When risk disclosure was experienced as *not* or *hardly burdensome*, participants had a positive self-perception (21.0%), utilized resources as coping mechanism (12.9%) and/or valued the possibility for informed decision-making (16.1%), e.g., *“It helps me to know that I am informed in good time”*.

### Consequences of risk disclosure

After learning about the increased risk of PD, 68.1% of participants did not notice any changes for the worse in their lives (Supplementary Table S2). Furthermore, the perceived physical condition remained unchanged for the majority (69.4%) and if it changed, only 35.5% attributed this to the recruitment study participation. Similarly, 62.5% reported an unchanged psychological state, 15.3% felt better, and 22.2% worse. If the state changed, 51.4% attributed this to the study participation.

Open-text responses (*n* = 71) revealed that 54.9% reported no consequences of risk disclosure on their life planning (Fig. 2D, Supplementary Table S3). Overall, when participants reported consequences, they were generally positively connoted, highlighting an increased focus on health through prevention and lifestyle changes (19.7%), e.g., in terms of an *“(…) even healthier lifestyle, more exercising (…)”*, or more mindful living (12.7%). One participant noted: *“Since the diagnosis, I have lived more intensively and consciously.”* Eight answers (11.3%) concerned environmental changes such as planning for early retirement *“to make the most of the time”*, organizing legal and healthcare matters, or *“no postponement of plans for desired trips any longer”*.

### Reasons for study participation

**Fig. 3:**
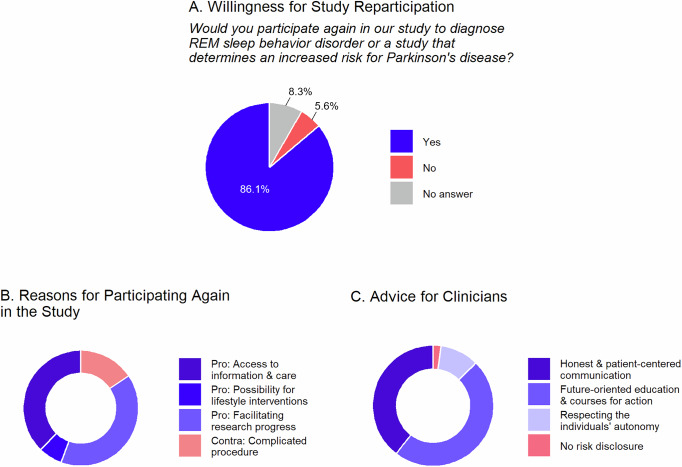
Qualitative reasons for participating again in the recruitment study and advice for clinicians. **A** Whether participants would be willing to participate again in the recruitment study. **B** Participants’ reasons for participating in the recruitment study. **C** Advice participants would like to give to clinicians regarding risk disclosure. Proportions in the donut chart correspond to the number of responses in each category. More information and exemplary quotes for each category can be found in Tables S4 and S5.

Despite the reported burden associated with risk disclosure, 86.7% would participate again in a study diagnosing iRBD or assessing the risk of PD (Fig. 3A). Among *n* = 47 open-question responses, 38.2% mentioned contributing to research progress as motivation for study participation, indicating a strong desire to advance medical care for those affected, even without any direct benefit to themselves (Fig. 3B, Supplementary Table S4). One participant shared: *“(…) If by participating in the study I can help to ensure that the disease is better diagnosed and above all better treated at some point, then it gives me a good feeling that I am doing something worthwhile.”* Others valued access to information and care (36.2%) and the possibility to implement lifestyle changes (6.4%, e.g., “*activities that help to improve (maintain) my health situation”*). One participant stated that *“I probably still wouldn’t know what I had”* without the recruitment study and reported getting *“examined very thoroughly”*. Argument against another study participation was the complicated procedure (14.9%), such as the time needed for the examination and traveling time.

Most participants felt they could decide for themselves whether they wanted to know about the increased risk (84.7%, Supplementary Table S2) and regarded it as *extremely* or *very* important to be fully informed, also on further diagnostic options, and to have a sufficient amount of time to clarify questions. Offering participation in further studies was not important for only 6.8%.

### Advice for clinicians

In response to the question on what advice participants would give clinicians when talking about an increased risk of Parkinson´s disease, we received a total of *n* = 49 comments. An honest, empathetic, and patient-centered communication was recommended by 38.8%, e.g., *“(..) address the matter openly (…)”* (Fig. 3D, Supplementary Table S5). Emphasizing the need to respect the individuals’ autonomy, one participant explained: *“Pay attention to what the patient wants or does not want, ask about it (…)”*. Also, 46.9% proposed future-oriented education and courses of action, e.g., “*Emphasize the positive aspect of early detection, address/offer targeted movement programs etc., present research results”*.

## Discussion

Latest progress in detecting α-synuclein in individuals with iRBD enables early biological identification of at-risk individuals for clinically overt α-synucleinopathies. Researchers are now testing disease-modifying therapies in clinical trials, which require active recruitment and identification strategies from the general population for those affected at an early stage. Therefore, we explored the experiences with risk disclosure of those affected and their perspectives on the ethical justifiability, using the example of an actively recruited iRBD cohort from the general population.

Almost all participants regarded risk disclosure as ethically justifiable if the participants’ autonomy was ensured. Regarding communication, participants emphasized the importance of honest, empathetic, and patient-centered conversations, focusing on offering support and providing actionable guidance. Views on the relevance of their sleep disorder before being enrolled in the active recruitment study varied; about a third worried and the majority highlighted the impact on their bed partners, while another third reported no burden, i.e., being in a ‘subjectively unaffected status’. The method of recruitment was widely accepted, though most experienced risk disclosure as burdensome. Participants reported diverse emotional and behavioral responses to risk disclosure, including worry, uncertainty, increased self-awareness, the use of coping mechanisms, and appreciation for the possibility of informed decision-making. The majority reported no lifestyle changes, others adopted more mindful habits, increased their health focus, or conducted environmental adjustments. Most participants would participate again in the recruitment study, naming benefits such as access to information and care, and the opportunity to contribute to research progress. With this work, we aimed to provide a guideline for active cohort recruitment in early α-synucleinopathy research, aligning with Schaeffer et al.’s recommendations on risk disclosure in prodromal PD and following the declaration of Helsinki^7,25^.

Although critical voices exist among individuals with PD as well as their clinicians^10,12^, our data elicits that individuals with iRBD—even in the absence of subjective burden—strongly favor risk disclosure. This is consistent with prior findings in individuals with iRBD and general population studies^16,17,26^. Similarly, participants receiving (positive) genetic testing for PD showed high satisfaction and individuals with PD believe it is right to inform individuals of a high PD risk if they wish to know^12,27^. This reinforces the ethical principle of autonomy: the individual’s right to make decisions, which implies obtaining informed consent before risk disclosure^28^.

Beyond autonomy, risk disclosure must consider the ethical principle of nonmaleficence^28^. Disclosing the risk of developing an overt α-synucleinopathy in the absence of an approved pharmacological disease-modifying therapy could lead to psychological distress^29^. However, our results show that, while most participants experienced burden, this was not linked to existing mental health factors, and the majority reported good psychological well-being. Additionally, most individuals reported no consequences of risk disclosure and appreciated access to information and care. This finding is in agreement with Alzheimer’s Disease research, that found no adverse psychological reactions to risk disclosure^30^.

Still, false positive diagnoses remain an ethical concern, even in the era of biomarkers^29^, underscoring the need to emphasize diagnostic uncertainty^25^. Additionally, social and political implications of diagnosis, such as stigma, should be considered^31^.

One of the most compelling arguments in favor of risk disclosure is the growing body of evidence suggesting that lifestyle interventions, particularly physical activity, can positively impact disease progression in (early) PD^32–34^. In line with the literature, individuals in our study showed interest in engaging in lifestyle interventions and studies^26^. A strong motivator for participation was the opportunity to contribute to research, mirroring findings in individuals with PD^35^.

Furthermore, early risk disclosure might reduce uncertainty, anxiety and misattribution of symptoms^29,30^. Our study showed that some individuals were bothered by their iRBD symptoms and in this case, early detection can avoid misdiagnosis while allowing rapid (symptomatic) treatment initiation that can maintain quality of life^11,12^. Also, knowledge opens the opportunity for life planning and could empower affected individuals to take proactive steps, further enhancing autonomy^31,36^. These considerations align with the principle of beneficence, which entails maximizing potential benefits^28^. Ultimately, actively withholding information is likely to destroy trust in the provider-patient relationship^16^.

Given this, the ethical debate may need to be reframed. Instead of questioning whether risk disclosure is ethically justifiable, one could ask whether it is justifiable *not* to inform affected individuals about their risk, thereby denying the opportunity to participate in disease-modifying intervention studies or performing lifestyle changes. When conducted responsibly, risk disclosure is not only justifiable but may be an ethical imperative.

Risk communication must be clear, accessible, and tailored to individual needs^25,29,37^. Our qualitative content analysis underscored the wish for honest, empathetic, and patient-centered communication that includes explaining the meaning of the diagnosis in layman’s terms. Offering diverse sources of information and allowing participants to choose their preferred risk disclosure approach fosters personalization^26^.

Furthermore, the risk disclosure setting should ensure time for questions^25^. A notable advantage of our research setting was the availability of extended time for consultations (~1.5 h), allowing for thorough discussions of individual questions. Also, we provided iRBD symptom management, further diagnostic options, consultation with neurologists, and participation in studies. In contrast, resources and consultation time in routine healthcare and some study settings are often significantly limited. We see a need for counseling in studies diagnosing early α-synucleinopathies.

Despite this study’s large sample size, the broad range of reported symptom duration, and the comprehensive mixed-methods approach, this study is limited by its geographically localized nature of the sample and its selection bias, as only individuals who actively sought information and were later diagnosed with iRBD participated in the survey. Furthermore, cultural factors may influence attitudes toward with risk disclosure^7,10^. Moreover, cognitive biases (e.g., cognitive dissonance, attentional or confirmation biases) could have affected ratings, autobiographic recall, or result in self-fulfilling prophecies^38^.

Future studies should investigate risk disclosure outcomes longitudinally across different cultures. Key questions remain on predicting specific phenotypes of α-synucleinopathies, discussing detailed prognoses with individuals, and handling risk disclosure in studies analyzing archived cerebrospinal fluid samples for α -synuclein^7^.

In summary, we argue in favor of risk disclosure upon active recruitment and suggest that diagnostic and, in particular, therapeutic studies in *uninformed* individuals should be viewed critically. As the recruitment strategy employed in this study was well-received by participants, the data suggest that our approach can serve as a model for an ethically sound, active recruitment of at-risk cohorts for α-synucleinopathy research. Emphasis should lay on an individually tailored approach to risk disclosure that ensures the individuals’ autonomy.

### Supplementary material

Tables S1 and S3 to S5 display themes and corresponding quotes from individuals with iRBD regarding the open-ended questions. Table S2 reports the frequencies for additional single-choice answers. Figure S1 shows the risk disclosure questionnaire sent to iRBD individuals. Figure S2 depicts a scatterplot illustrating the combination of PHQ-9, GAD-7 and BRS scores for all individuals separated by sex.

## Supplementary information

Supplementary information

## Data Availability

Anonymized data are available at a reasonable request to the corresponding author.
