# Supplementary material for: The prodromal individuals’ perspective on active recruitment for early detection of α-synucleinopathies
Source: NPJ Parkinsons Dis. 2025 May 9;11:121. doi: 10.1038/s41531-025-00979-0 (PMC12064827; doi:10.1038/s41531-025-00979-0)
Supplement: Supplementary file 1 — Supplementary information [file 41531_2025_979_MOESM1_ESM.pdf]

Supplementary Material

Table of Contents

- Table S1. Themes and corresponding quotes from individuals with iRBD regarding the questions: “How did you experience the information about the increased risk? Can you give reasons for this evaluation?”
- Table S2. Frequencies for additional single-choice answers.
- Table S3. Themes and corresponding quotes from individuals with iRBD regarding the question: “What consequences did the information about the increased risk have for you personally? Did it have an influence on your personal life planning?”
- Table S4. Themes and corresponding quotes from individuals with iRBD regarding the question: “Would you participate again in our study to diagnose REM sleep behavior disorder or a study that determines an increased risk for Parkinson's disease?”
- Table S5. Themes and corresponding quotes from individuals with iRBD regarding the question: “What advice would you give to clinicians (doctors, nurses and other healthcare professionals) when talking to people about an increased risk of Parkinson's disease?”
- Figure S1. Mixed-method questionnaire on risk disclosure preferences in an active cohort recruitment setting for individuals with isolated REM sleep behavior disorder.
- Figure S2. Scatterplot illustrating the combination of PHQ-9, GAD-7 and BRS scores for all individuals separated by sex.

Table S1. Themes and corresponding quotes from individuals with iRBD regarding the questions: "How did you experience the information about the increased risk? Can you give reasons for this evaluation?"

**(Positive) self-perception** (n=13 answers)	**Utilizing resources** (n=8 answers)	**Value of informed decision-making** (n=10 answers)	**Experiences of worries & uncertainty** (n=26 answers)
"(...One) observes oneself more"	*"Think positively, do not burden yourself beforehand"*	*"It helps me to know that I am informed in good time."*	*"Disease is not curable"*
"I am almost seventy [years old] and still have no symptoms, and I assume that this will continue for a long time to come"	*"After (…) researching on the internet, one is already informed!"*	*"With the information [about the risk], I can better organize my further actions."*	*"I had to deal with the thought of a serious illness in the not-too-distant future."*
"So far (subjectively) no impairments, therefore optimistic […]. The positive thing for me is that one may be able to do something to counteract the possible course of the disease, which will at least delay its progression." [a]	*"I am a devout Christian and know that I am in God's hands."*	*"On the one hand, it was a burden for me to have an increased risk […], but on the other hand, it does not have to affect me. The positive thing about my [iRBD] diagnosis was that I was able to put a name to my nocturnal excesses. They no longer affect my personal self-esteem."*	*"The decisive factor is the unknown as to when and to what extent deterioration will occur..."*

In total, *N* = 62 participants responded to this open question. Three answers were grouped into two categories each. Eight answers were not categorizable and suggested a misunderstanding of the question.

[a] answer also grouped into the category "value of informed decision-making".

Table S2. Frequencies for additional single-choice answers.

Question	Response option	*n*	%
Q8. Life changes (better) after risk disclosure	Yes, my life has changed somewhat for the better	25	34·7%
	I do not know	18	25·0%
	No, nothing has changed for the better in my life	29	40·3%
Q9. Life changes (worse) after risk disclosure	Yes, my life has changed somewhat for the worse	13	18·1%
	I do not know	9	12·5%
	No, nothing has changed for the worse in my life	49	68·1%
	No answer	1	1·4%
Q10. Physical condition	Unchanged	50	69·4%
	Changed: Better than before	13	18·1%
	Changed: Worse than before	9	12·5%
Q11. If changed: Change due to study?	Yes	11	35·5%
	No	20	64·5%
Q12. Psychological state	Unchanged	45	62·5%
	Changed: Better than before	11	15·3%
	Changed: Worse than before	16	22·2%
Q13. If changed: Change due to study?	Yes	18	51·4%
	No	17	48·6%
Q16. Autonomous decision	Completely true	33	45·8%
	Mainly true	28	38·9%
	Less true	7	9·7%
	Does not apply at all	2	2·8%
	No answer	2	2·8%
Q17. Importance of information	Extremely important	36	50·0%
	Very important	36	50·0%
Q18. Importance of time to ask questions	Extremely important	32	44·4%
	Very important	39	54·2%
	Less important	1	1·4%

Q19. Importance of information on further diagnostic options	Extremely important	41	56·9%
	Very important	27	37·5%
	Less important	3	4·2%
	No answer	1	1·4%
Q20. Importance of study participation offers	Extremely important	26	36·1%
	Very important	41	56·9%
	Not at all important	5	6·9%

N=72. Results are expressed as absolute numbers, %.

Table S3. Themes and corresponding quotes from individuals with iRBD regarding the questions: “What consequences did the information about the increased risk have for you personally? Did it have an influence on your personal life planning?”

**No consequences** (n=39 answers)	**Setting environmental adjustments** (n=8 answers)	**Increased Health focus** (n=14 answers)	**More mindful living** (n=9 answers)
“Not really, as I have not been able to detect any symptoms so far.”	*“Considering taking early retirement to make the most of the time”*	*“Yes, it has strengthened my willingness to participate in further studies.”*	*“Since the diagnosis, I have lived more intensively and consciously.”*
“It has no influence on my life planning. I think I have a solid life and am generally positive about the future [...]”	*“Increased sensitivity and thoughtfulness regarding health care proxies, arrangements for estate/legacy, information for family”*	*“Personal life planning has basically not changed so far, apart from the need to pay even more attention to personal fitness and exercise/endurance sports.”*	*“Conscious, grateful dealing with the healthy now state. Careful dealing with the REM sleep behavior disorder.”*
“Not yet, may change with the first symptoms”	*“No postponement of plans for desired trips any longer, even healthier lifestyle, more exercise, further participation in studies”* [a]	*“My life plan has not changed, at most I have been encouraged to intensify my cognitively, coordinatively and physically demanding hobbies. However, I have to consciously refrain from overestimating possible symptoms due to the increased risk.”*	*“The time that remains for me and my wife will now be lived even more lively than before. Thank you”*

In total, *N* = 71 participants responded to this open question. One answer was grouped into two categories each. Three answers were not categorizable and suggested a misunderstanding of the question.
[a] answer also grouped into the category “increased health focus”.

Table S4. Themes and corresponding quotes from individuals with iRBD regarding the question: "Would you participate again in our study to diagnose REM sleep behavior disorder or a study that determines an increased risk for Parkinson's disease? Please explain your answer."

**Pro: Access to information & care** (n=17 answers)	**Pro: Possibility for lifestyle interventions** (n=3 answers)	**Pro: Facilitating research progress** (n=18 answers)	**Contra: Complicated procedure** (n=7 answers)
"I generally find such studies very important; I personally benefit from them in order to be better informed myself"[a]	*"I will do all activities that help to improve (maintain) my health situation, also with a view to the future."*	*"I am happy to do this if it helps research. I or other people may benefit from the findings collected and evaluated"*	*"I didn't realize how much time [examinations and traveling] would take […]"*
"iRBD has had a very negative impact on my life over the past few years. I am glad that it has now been clarified what the nocturnal losses are […]. And as I don't want to run into the dark, I will also take part in all further examinations to find out whether I already have Parkinson's and how far it has progressed."	*"I am glad to know about the increased risk at an early stage […]. I also enjoy the "prophylactic measures" that I do, so I make time for them."*[b]	*"1. I like to know what I'm dealing with so that I can determine my own options for action. 2. If by participating in the study I can help to ensure that the disease is better diagnosed and above all better treated at some point, then it gives me a good feeling that I am doing something worthwhile."*[b]	*"I am unsure because I wanted to find out about the behavioral disorders, especially what could be done to improve my condition, now it´s more about Parkinson's"*
"I probably still wouldn't know what I had and I get examined very thoroughly."	*"[…] More conscious involvement and exercise in all aspects of life and activities of any kind."*	*"To help other affected people at an early stage in the future."*	*"[…] confirming the diagnosis is very cumbersome and […] there is no effective remedy […]"*

In total, *N* = 47 participants responded to this open question. Seven answers were grouped into two categories each. Nine answers were not categorizable and suggested a misunderstanding of the question.
[a] answer also grouped into the category "facilitating research progress" [b] answer also grouped into the category "access to information & care".

Table S5. Themes and corresponding quotes from individuals with iRBD regarding the question: "What advice would you give to clinicians (doctors, nurses and other healthcare professionals) when talking to people about an increased risk of Parkinson's disease?"

**Honest & patient-centered communication** (n=19 answers)	**Respecting the individuals' autonomy** (n=5 answers)	**Future-oriented education & courses for action** (n=23 answers)	**No risk disclosure** (n=1 answer)
"Deal openly with the disease and speak about it."	*"Pay attention to what the patient wants or does not want, ask about it and take it into account. Only have such conversations if your own attitude is truly empathetic. Take your time."* [a]	*"Advise patients to pay attention to symptoms, specify a time frame for check-ups, advise on behavior if possible"*	*"Looking back, I wouldn't bring it up. I don't see the point."*
"Be honest and explain in layman's terms what the diagnosis may mean"	*"Find out beforehand whether the person wants to know."*	*"Showing the way to counter Parkinson's disease, presenting mitigation through medical measures"*	
"Always address the matter openly and specifically, but this always depends on the person receiving the diagnosis."	*"The person must want the conversation themselves!"*	*"Emphasize the positive aspect of early detection, address/offer targeted movement/exercise programs etc., present research results"*	

In total, *N* = 49 participants responded to this open question. Seven answers were grouped into two categories each. Eight answers were not categorizable (e.g., *"I don't know"*).
[a] answer also grouped into the category "honest & patient-centered communication"

In the following, we would like to ask you a few questions on the subject of “REM sleep behavior disorder” and the risk information risk information provided by us in this context.

Q1: How relevant did you consider your sleep disorder until you contacted us?

Multiple selection possible:

- It didn't bother me
- I was worried
- My bed partner was disturbed
- I have already hurt myself once or several times in my sleep
- I have hurt my bed partner one or more times
- I cannot/do not want to answer this question

For our study, we used newspaper advertisements to search for people with REM sleep behavior disorder. You were diagnosed with REM sleep behavior disorder through the sleep assessment.

In the course of this, we spoke to you about the increased risk of developing Parkinson's disease in the future.

Q2: Do you think it is okay to inform people that they have a high risk of developing Parkinson's disease risk in the course of their lives, even before they show clear signs of the disease?

Multiple selection possible:

- No, never
- Yes, always
- Yes, but only if this leads to new insights for the treatment of other patients
- Yes, but only if this leads to new treatment options for those affected
- Yes, but only if it has been ensured beforehand that the person concerned wants to know the risk
- I cannot/do not wish to answer this question

Q3: Do you think it is okay for people to be made aware of their increased risk of Parkinson's disease through a study like the one we are conducting?

- I think it's absolutely fine
- I think it is mostly okay
- I think it's rather not okay
- I don't think it's okay at all
- I cannot/do not want to answer this question

Q4: How did you experience the information about the increased risk?

- Not burdensome
- Hardly burdensome
- Somewhat burdensome
- Very burdensome
- I cannot/do not want to answer this question

Q5: Can you give reasons for this assessment?

Free text (optional):

Q6: What consequences did the information about the increased risk have for you personally? Did it influence your personal life planning?

Free text (optional):

Q7: What advice would you give to clinicians (doctors, nurses and other healthcare professionals) when talking to people about an increased risk of Parkinson's disease?

Free text (optional):

Q8: Has anything changed for the better in your life as a result of learning about the increased risk of Parkinson's in the context of REM sleep behavior disorder?

- Yes, my life has changed somewhat for the better
- I do not know
- No, nothing has changed for the better in my life
- I cannot/do not want to answer this question

Q9: Has anything changed for the worse in your life as a result of being told about the increased risk of Parkinson's in the context of REM sleep behavior disorder?

- Yes, something has changed for the worse in my life
- I do not know
- No, nothing has changed for the worse in my life
- I cannot/do not want to answer this question

Q10: How would you describe your physical condition since you have been involved in our study?

- Unchanged
- Changed: Better than before
- Changed: Worse than before
- I cannot/do not want to answer this question

Q11: If changed: In your opinion, does this change have anything to do with your participation in the

study?

- Yes
- No
- I cannot/do not want to answer this question

Q12: How would you describe your psychological state since you have been involved in our study?

- Unchanged
- Changed: Better than before
- Changed: Worse than before
- I cannot/do not want to answer this question

Q13: If changed: In your opinion, does this change have anything to do with your participation in the

study?

- Yes
- No
- I cannot/do not want to answer this question

Q14: Would you participate again in our study to diagnose REM sleep behavior disorder or a study that determines an increased risk for Parkinson's disease?

- Yes
- No
- I cannot/do not want to answer this question

Q15: Please explain your answer.

Free text (optional):

Q16: Did you feel that you were able to decide for yourself whether you wanted to know about an increased risk of developing Parkinson's disease as part of our study?

- Completely true
- Mainly true
- Less true
- Does not apply at all
- I cannot/do not want to answer this question

Q17: How important is it to you that you are kept fully informed?

- Extremely important
- Very important
- Less important
- Not at all important
- I cannot/do not want to answer this question

Q18: How important is it to you that you are given enough time to ask your own questions?

- Extremely important
- Very important
- Less important
- Not at all important

- I cannot/do not want to answer this question

Q19: How important is it to you that you are given sufficient information about further diagnostic options?

- Extremely important
- Very important
- Less important
- Not at all important
- I cannot/do not want to answer this question

Q20: How important is it to you that participation in scientific studies is offered?

- Extremely important
- Very important
- Less important
- Not at all important
- I cannot/do not want to answer this question

Figure S1. Mixed-method questionnaire on risk disclosure preferences in an active cohort recruitment setting for individuals with isolated REM sleep behaviour disorder.

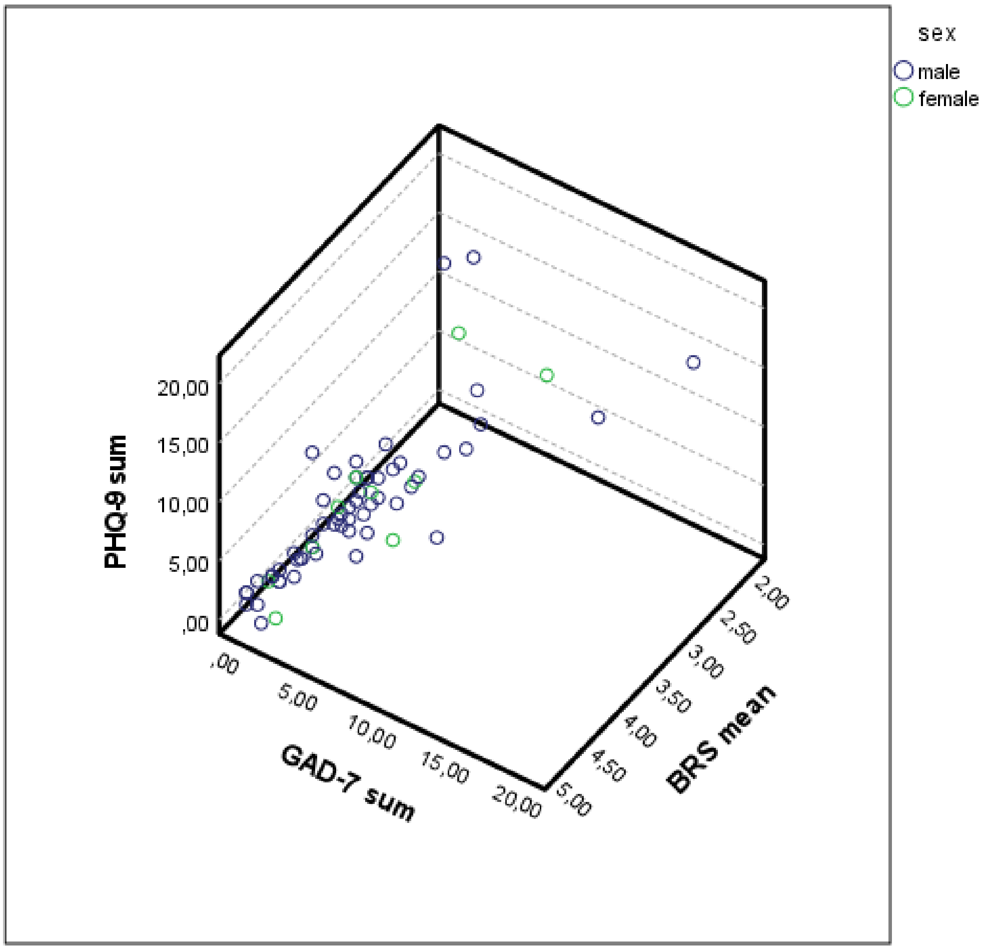

Figure S2. Scatterplot illustrating the combination of PHQ-9, GAD-7 and BRS scores for all individuals separated by sex. N=72.
